# How mothers feel: Validation of a measure of maternal mood

**DOI:** 10.1111/jep.13304

**Published:** 2019-11-25

**Authors:** Emily Savage McGlynn, Colin R. Martin, Maggie Redshaw

**Affiliations:** ^1^ Policy Research Unit in Maternal Health and Care, National Perinatal Epidemiology Unit University of Oxford Oxford UK

**Keywords:** adjective checklist, maternal mood, maternal mood checklist, mental health, perinatal

## Abstract

**Rationale:**

Low mood may affect developing relationships with a new baby, partner and family. Early identification of mood disturbance is crucial to improve outcomes for women perinatally. Instruments such as the Edinburgh Postnatal Depression Scale (EPDS) are used routinely, with evidence that some women do not feel comfortable with how they are asked about their mental health.

**Objective:**

To develop a mood checklist as a user‐friendly, effective measure of well‐being in post‐partum women, for use by health professionals.

**Methods:**

Cognitive interviews with women who had recently given birth assessed response format and face validity of a prototype measure. A cross‐sectional survey followed. A random split‐half instrument development protocol was used. Exploratory factor analysis determined factor structure with the first sample,. The second sample confirmed factor structure and evaluationof key psychometric variables and known‐groups discriminant validity (KGDV), requiring a supplementary between‐subjects design with stratification based on case negative/case positive classification using EPDSscreening cut‐off criteria.

**Results:**

Cognitive interview data confirmed the face validity of the measure. Exploratory factor analysis indicated an 18 item two‐factor model with two (negatively) correlated factors. Factor 1 loaded with items reflecting positive mood and factor 2 negative items. Confirmatory factor analysis showed a good fit to the two‐factor model across the full spectrum of fit indices. Statistically significant differences between groups were observed in relation to as EPDS caseness classification. Cronbach alpha coefficients for the positive and negative subscales revealed acceptable internal consistency of 0.79 and 0.72, respectively.

**Conclusion:**

The outcome checklist may be appropriate for use in clinical practice. It demonstrated effective psychometric properties and clear cross‐validation with existing commonly used measures.

## 1 INTRODUCTION

Mood disorders are known to be common among women during the perinatal period. Studies estimate the point prevalence between 8% and 15%,^1^, ^2^ but it has been argued that this is an under‐representation of the true scale of incidence.^3^ Larger proportions have been reported when interval data are used.^2^ Among the reasons responsible for under‐representation of identification of mood disturbances during the perinatal period include a lack of willingness of women to divulge concerns with their mental health to a health care professional and a lack of continuity of care fundamental to develop rapport with a trusted health care provider.^4^ Current screening instruments and assessments may not be perceived by women as suitable or appropriate ways for them to report their true feelings during this time. The Edinburgh Postnatal Depression Scale (EPDS) is a commonly used screening measure for symptoms of depression during the perinatal period.^2^ However, the scale itself can be perceived as judgmental by women, with universally negatively structured items.^5^, ^6^ Women have reported the item wording to be leading and obvious to the nature of the scale, hence easy to cover up their true feelings should they wish to do so. It has been argued that the scale used as a whole is focused on depressive symptoms, rather than mood that is a more nuanced construct.^7^, ^8^ It has also been suggested that the subscales relating to depression and anxiety should be used separately, for both research and screening processes along with other measures.^9^, ^10^, ^11^

Widely used mood adjective checklists such as the Positive and Negative Affect Schedule (PANAS)^12^, ^13^ and the Scale of Positive and Negative Experiences (SPANE)^14^ have not been validated with a perinatal population. Consequently, these may not be effective in assessing the full scope of the mood differences and the health and well‐being issues facing women at this time in their lives. They have a potential for skewed findings arising from symptoms common during the perinatal period that could be misinterpreted as mood such as fatigue, lethargy, and loss/increase of appetite. Some adjective checklists have been reported to have been used successfully with women during pregnancy, although these were not specifically designed for this group and sample sizes were fairly small, an acknowledged issue among the study limitations.^15^, ^16^ In these studies, researchers have, for example, compared the well‐being of women having in vitro fertilization (IVF) treatment compared with that of women conceiving normally and investigated whether women with recurrent implantation failure after IVF are similar in this regard to women with recurrent pregnancy loss following natural conception. Both groups experienced higher stress levels compared with women without reproductive failure. These latter examples both used the PANAS, which has largely been validated on young adults, most commonly the student population,^12^ although some community samples of men and women aged between 18 and 91 years have been studied.^10^, ^13^ It has been also used but in a modified form, with younger clinical psychiatric populations and very disadvantaged groups.^17^, ^18^

All of the commonly used checklists so far described have employed positive and negative term with graded Likert‐type responses required and the duration of time over which respondents describe themselves varies from “today” or “the present moment” and “the past week” to “past four weeks” or “past month.”^12^, ^14^ The past week has been used with clinical populations^19^ as with a variety of other non‐checklist measures such as the EPDS.^20^ Substantially longer adjective checklists have been used in the past in studies of current state, with a focus on links with physiological factors, stress, and arousal.^21^, ^22^, ^23^

However, for women in the perinatal period, a simpler checklist has high face validity as evidenced by responsiveness in large‐scale studies with two simple checklists that were validated for use in the post‐natal period, describing care during labour and birth^24^, ^25^ and how mothers perceived their young infants.^26^, ^27^ A need for an easily administered tool for assessing maternal mood in the perinatal period was identified as part of a programme of work looking at women's maternity experiences. The objective was thus to develop a checklist measure of maternal mood using a limited response format that is easy for women to complete, simple to score and interpret, which reflects the range of emotions experienced by women in the perinatal period and that correlates with the data collected using a standard diagnostic screening tool. Using a population‐based survey, the present study aimed to develop and then determine the factor structure and validity of a maternal mood checklist (MMCL).

## 2 METHODS

A review of the literature was conducted to identify commonly used measures and checklists with diverse populations, including women of reproductive age and with perinatal populations, if any. A review was also undertaken of the adjectives reported and terms actually used to express their mood by women in the free text sections of the National Maternity Surveys from 2010 and 2014 where they wrote about their experience of maternity care and the early months at home with a new baby.^28^ Scoping of the measure was undertaken, and a preliminary list of 24 items was drafted for psychometric evaluation and data reduction. Cognitive “think‐aloud” interviews focusing on the possible items and women's concurrent feelings and mood were then conducted and audio recorded with nine women who had given birth within the last 3 months. The interviewees were also asked about other terms they might additionally use to describe how they felt.

### 2.1 Design

The aim with this checklist was to develop a measure of maternal mood (the MMCL) and to test a simpler structure and response format with terms that were of direct relevance to pregnant and post‐natal women. Potential descriptor terms were selected and tested in the cognitive interviews. Simple formatting aimed to encourage response, reduce the burden to participants, and facilitate scoring. A binary scoring system (endorsed/non‐endorsed) was utilized with women selecting adjective target words by circling these, as with previously described checklists.^24^ Non‐endorsement was indicated by the absence of a marked adjective. Consequently, item‐level scoring was “0” for non‐endorsement and “1” for endorsement. Subscales identified within the measure would produce total subscale scores. In view of the changes over time of women's mood following childbirth, “the last 7 days” was chosen for use with the new measure.

### 2.2 Participants

In October 2016, as part of a pilot for national survey of post‐natal maternal and child health and care in England, the mood adjective checklist was included for the sample of recent new mothers. Women were selected randomly by the Office for National Statistics (ONS) from birth registration records for births (N = 2000). Stratification of the sample was based on births in different geographical areas (Government Office Regions). Women experiencing a perinatal loss and young mothers less than 16 years of age were excluded. The ONS mailed the survey months using a tailored reminder system at either 3 or 6 months after the birth.

### 2.3 Ethical approval

Ethical approval for the cognitive interviews was obtained from Oxford University Medical Sciences Interdivisional Research Ethics Committee (IDREC R46227/RE001). For the survey of recent mothers, approval was granted by the NRES committee for Yorkshire and The Humber – Sheffield Research Ethics Committee (REC reference 16/YH/0412).

The study used a two‐stage cross‐sectional design comprising a random split‐half instrument development and testing protocol.^29^, ^30^, ^31^ The first split‐half data set (data set 1) was used to determine underlying factor structure and the second split‐half data set (data set 2) to confirm factor structure and evaluate the MMCL for key psychometric properties.

### 2.4 Statistical analysis

In preparation, prior to splitting the data set, potential MMCL and key scale‐based data parameters used to evaluate psychometric properties were screened for accuracy, missing data, distributional normality, and outliers. Kline^30^ suggests that skew values greater than 3 and kurtosis greater than 10 indicate non‐normality. Mahalanobis distances were used to determine multivariate outliers, a threshold chi square calculated (*P* < .001) and those cases beyond threshold eliminated (n = 8).

Exploratory factor analysis (EFA) was used with data set 1 to determine underlying factor structure and identify poorly performing and cross‐loading items. The principal axis factoring (PAF) factor extraction procedure was selected consistent with binary response categorzation of the MMCL^32^ and with factor analytic approaches to non‐normal data.^33^ Identification of the number of factors for extraction was aided by parallel analysis^34^ and scrutiny of Cattell scree plot.^35^ Anticipating that underlying factors were likely to be correlated, the oblimin method of rotation of extracted factors was selected.^32^ Initial identification of significant item‐factor loadings was based on a coefficient criterion of.30 or greater.

Confirmatory factor analysis (CFA) was used with data set 2 to evaluate and confirm the factor structure identified by EFA in data set 1.^30^ Consistent with the approach taken with data set 1, the multivariate and univariate normality characteristics of data set 2 were evaluated prior to the CFA.^35^, ^36^ Diagonally weighted least squares (DWLS) was used to estimate model parameters. This approach to model evaluation is consistent with data that are distributionally non‐normal and binary/categorical.^37^ Multiple goodness of fit tests^38^ were used to evaluate the models: comparative fit index (CFI) values greater than 0.90 indicate an acceptable data fit, values of 0.95 a good fit^39^, ^40^; root mean squared error of approximation (RMSEA) values of less than 0.05 indicate a good fit to the data^41^; the weighted root mean square residual (WRMR) values of less than 1.00 indicate acceptable model fit and 0.05 or less a good fit.^38^ Squared root mean square residual (SRMR) values of less than 0.08 indicate adequate fit and values of 0.05 or less good fit to data.^39^, ^40^, ^41^, ^42^, ^43^ The WRMR is used in the EFA and the SRMR in the CFA.

Divergent validity of MMCL subscale scores was determined by examination of the correlation (Spearman rho) between these scores and the number of weeks pregnant at the time of delivery. It was predicted that there would be no significant relationship between MMCL subscale scores and this clinical parameter.

Estimation of convergent validity was conducted by correlating MMCL subscale scores with the Oxford Worries about Labour Scale (OWLS)^44^ score. The OWLS is a 9‐item validated self‐report measure of prior worry about labour and birth, higher scores on the OWLS indicating comparatively *less* worry. It was predicted that MMCL subscales would be significantly correlated (Spearman rho) with the OWLS total score.

Known‐groups discriminant validity was evaluated by determining MMCL subscale score differences as a function of post‐natal depression status as determined by the EPDS^20^ using the non‐parametric Mann‐Whitney U test. The threshold for clinically significant caseness based on EPDS score was 12/13 (case negative/case positive). It was predicted that there would be statistically significant differences in MMCL subscale scores as a function of EPDS caseness categorization.

The internal consistency characteristics of the MMCL was determined using Cronbach coefficient alpha. An alpha of.70 or greater is considered acceptable.^30^ Statistical analysis was conducted using the statistical software package R.^45^

## 3 RESULTS

### 3.1 Cognitive interview results

When the nine women participating in the think‐aloud interviews were asked if there were any other terms they might additionally use to describe how they felt, “tired” was most commonly mentioned; however, as a somatic symptom, this was not included in the list. Terms that generally although not specifically reflected positive mood were suggested by a few mothers, for example, “lucky,” “loved,” and “grateful” but were not included. The women emphasized the importance of being able to respond positively and negatively about their mood, without “having to think hard” about a graded response, and being able to choose just the terms they felt applied to them. They also reported positively about their diverse and often mixed feelings being normalized by being given a range of potential mood descriptors from which to choose. Feedback on the structure and content supported the key elements of the design and format.

### 3.2 Descriptive results

A total of 504 women returned usable data by postal questionnaire in this pilot survey with a 28% response rate. Complete MMCL data were available on 488 participants (approximately 3% missing data). Seven multivariate outliers were detected, and these cases removed from the data set, leaving a final N = 481 (approximately 1.5% outlier removal). The mean age of participants was 31.93 (*SD*, 5.55) years, a total of 87% self‐identified as being from a White ethnic background and 90% were living with their spouse or partner. The average duration of pregnancy was 39.00 weeks. For half this was their first baby (48%), and almost all women (96%) had a single baby. The majority (92%) had their baby in hospital, with half (49%) having their baby delivered in an alongside or free‐standing midwifery led unit.

The random‐split procedure produced an EFA data set of N = 240 and a CFA data set of N = 241.

Means, standard deviations, skew, and kurtosis of MMCL items for data set one are summarized in Table 1. Examination of skew and kurtosis characteristics suggested no evidence of substantive deviation from a univariate normal distribution (skew, less than 3; kurtosis, less than 10) with the exception of item 4. “Detached” demonstrated excessive kurtosis and a minimal level of endorsement (less than 5%). This item was consequently excluded from the instrument.

**Table 1:** Mean, standard deviation, distributional characteristics, and endorsement status of MMCL items

Item	Mean	*SD*	Skew	Kurtosis	−ve	+ve
Calm	0.46	0.50	0.15	−1.99	129	111
Irritable	0.35	0.48	0.61	−1.64	155	85
Confident	0.39	0.49	0.44	−1.81	146	94
Detached	0.06	0.24	3.67	11.50	230	10
Cheerful	0.51	0.50	−0.03	−2.01	118	122
Restless	0.15	0.36	1.90	1.63	203	37
Tense	0.24	0.43	1.20	−0.56	182	58
Happy	0.74	0.44	−1.07	−0.85	63	177
Drained	0.43	0.50	0.28	−1.93	137	103
Contented	0.47	0.50	0.12	−1.99	127	113
Angry	0.12	0.32	2.37	3.65	212	28
Lighthearted	0.14	0.35	2.09	2.36	208	32
Relaxed	0.41	0.49	0.35	−1.88	141	99
Miserable	0.09	0.29	2.81	5.94	218	22
Fulfilled	0.35	0.48	0.63	−1.62	156	84
Low	0.16	0.37	1.82	1.31	201	39
Energetic	0.16	0.37	1.86	1.47	202	38
Worried	0.27	0.45	1.02	−0.95	175	65
Upset	0.20	0.40	1.46	0.13	191	49
Excited	0.25	0.43	1.17	−0.63	181	59
Nervous	0.18	0.38	1.66	0.77	197	43
Optimistic	0.35	0.48	0.64	−1.59	157	83
Impatient	0.18	0.38	1.70	0.89	198	42
Satisfied	0.37	0.48	0.53	−1.72	151	89

Abbreviation: MMCL, maternal mood checklist.

### 3.3 Exploratory factor analysis

The Kaiser‐Meyer‐Olkin measure of sampling adequacy (0.82) and the Bartlett test of sphericity (*χ* ^2^ = 1301.5, *df* = 253, *P* < .001) indicated data set 1 was appropriate for EFA. Examination of the scree plot and parallel analysis indicated a three‐factor solution. Three correlated factors were extracted with eigenvalues of 4.84, 2.87, and 1.33 explaining 39% of the variance. However, two cross‐loading items were noted: “tense” and “angry.” The EFA was then rerun excluding these two items and again; three correlated factors were observed with eigenvalues of 4.50, 2.45, and 1.31, explaining 39% of the variance. It was noted that the third factor comprised just three items and as a subscale demonstrated poor internal consistency (Cronbach alpha = .54). To pursue parsimony, these three items, namely, “irritable,” “drained,” and “impatient,” were rejected and removed in favour of a two‐factor model specification.

This two‐factor model was run, revealing two (negatively) correlated factors with eigenvalues of 4.30 and 2.16 explaining 36% of the variance and comprising 18 items. Factor 1 is loaded with items representing positive items of mood, and factor 2 conversely with those representing negative aspects. Model fit was found to be good, *χ* ^2^(*df* = 118) = 15 499, *P* = .01, CFI = 0.95, TLI = 0.93, RMSEA = 0.04 (90% CI, 0.02‐0.05), RMSR = 0.05. The item‐factor loadings are summarized in Table 2.

**Table 2:** Factor loadings of the maternal mood MMCL following PAF exploratory factor analysis (split‐half data set, N = 240)

Item	Factor 1	Factor 2
Calm	**0.6**	−0.02
Confident	**0.58**	−0.04
Cheerful	**0.65**	0.03
Restless	−0.05	**0.37**
Happy	**0.50**	−0.12
Contented	**0.38**	−0.11
Lighthearted	**0.36**	0.06
Relaxed	**0.6**	−0.05
Miserable	−0.01	**0.67**
Fulfilled	**0.45**	−0.08
Low	−0.06	**0.61**
Energetic	**0.37**	0.04
Worried	−0.05	**0.58**
Upset	0.06	**0.61**
Excited	**0.56**	0.23
Nervous	0.04	**0.42**
Optimistic	**0.37**	−0.06
Satisfied	**0.50**	−0.09

Abbreviations: MMCL, maternal mood checklist; PAF, principal axis factoring.

*Note*: Bold emphasis shows the factor loadings clearly as required.

### 3.4 Data set 2 distributional characteristics

The means, standard deviations, skew, and kurtosis of data set 2 MMCL items are summarized in Table 3. None of the items exhibit any indication of undue skew or kurtosis with the sole exception of the item “miserable,” which demonstrates marginally excessive skew. The mean of the 12‐item positive item subscale (CL‐P) was 4.71 (*SD*, 3.05), and for the 6‐item negative subscale (CL‐N), it was 1.06 (*SD*, 1.46)

**Table 3:** Mean, standard deviation and distributional characteristics of MMCL items in split‐half confirmatory factor analysis data set (N = 241)

Item	Mean	*SD*	Skew	Kurtosis
Calm	0.52	0.50	−0.09	−2.00
Confident	0.41	0.49	0.38	−1.86
Cheerful	0.52	0.50	−0.07	−2.00
Restless	0.17	0.38	1.71	0.92
Happy	0.76	0.43	−1.21	−0.55
Contented	0.45	0.50	0.21	−1.97
Lighthearted	0.13	0.34	2.15	2.64
Relaxed	0.38	0.49	0.50	−1.76
Miserable	0.07	0.26	3.22	8.37
Fulfilled	0.32	0.47	0.79	−1.38
Low	0.20	0.40	1.47	0.15
Energetic	0.19	0.39	1.56	0.45
Worried	0.27	0.44	1.03	−0.94
Upset	0.20	0.40	1.50	0.24
Excited	0.27	0.45	1.01	−0.99
Nervous	0.14	0.34	2.10	2.42
Optimistic	0.34	0.47	0.69	−1.53
Satisfied	0.44	0.50	0.26	−1.94

Abbreviation: MMCL, maternal mood checklist.

### 3.5 Confirmatory factor analysis

CFA was conducted on data set 2 specifying the final two‐factor model derived from the EFA. A single‐factor version of this model was estimated for comparative purposes. A good fit to the two‐factor model was found across the full spectrum of fit indices. The alternative single‐factor model offered a generally poorer fit to data. The model fit characteristics of both models are summarized in Table 4.

**Table 4:** Evaluation of the structure of the MMCL by CFA using the second split‐half data set (N = 241)

Model	DWLS (*χ* ^2^)	*df*	*P*	CFI	TLI	RMSEA (90% CI)	SRMR
1. **Two factors**	164.7	134	.04	0.98	0.97	0.03 (0.01‐0.05)	0.06
2. Single factor	288.6	135	.001	0.89	0.87	0.07 (0.06‐0.08)	0.10

*Note*. Best model fit from confirmatory factor analysis indicated in bold.

Abbreviations: CFA, confirmatory factor analysis; CFI, comparative fit index; DWLS, diagonally weighted least squares; MMCL, maternal mood checklist; RMSEA, root mean squared error of approximation; SRMR, standardized root mean residual; TLI, Tucker–Lewis index.

### 3.6 Divergent validity

No significant correlation was observed between both MMCL subscales and the number of weeks pregnant at delivery (CL‐P subscale, *r*_s_ *=* .06, *P* = .34; CL‐N subscale, *r*_s_ *=* −.07, *P* = .26).

### 3.7 Convergent validity

Statistically significant correlations were found between both CL‐P and CL‐N subscales and the OWLS worry scale (CL‐P subscale, *r*_s_ *=* .15, *P* = .02; CL‐N subscale, *r*_s_ = −.20, *P* < .01).

### 3.8 Known‐groups discriminant validity

Mean CL‐P and CL‐N subscale scores and results of statistical evaluation are summarized in Table 5. Mean EPDS scores for the caseness negative group were 5.38 (*SD*, 3.26) and 16.58 (*SD*, 3.24) in the caseness positive group. Highly statistically significant differences between groups were observed as a function EPDS this classification. CL‐P subscale scores were significantly higher in the EPDS caseness negative group (median = 5) compared with the positive group (median = 2). CL‐N subscale scores were significantly higher in the EPDS positive group (median = 3) compared with the negative group (median = 0).

**Table 5:** Mean MMCL subscale scores as a function of EPDS case categorization (non‐depressed N = 196, depressed N = 38). (Standard deviations in parentheses)

Variable	Nondepressed	Depressed	Mann–Whitney U	*P*
CL‐P	5.21 (2.91)	2.15 (2.39)	5939	<.001
CL‐N	0.74 (1.20)	2.79 (1.56)	1068	<.001

*Note*. Seven missing cases are due to incomplete EPDS data. Standard deviations are in parentheses.

Abbreviations: EPDS, Edinburgh Postnatal Depression Scale; MMCL, maternal mood checklist.

### 3.9 Internal consistency

Given that the final MMCL measure comprised two *negatively* correlated subscales, the use of a total score was deemed inappropriate, and consequently, a total score of alpha was not calculated since several items would be highly negatively and positively correlated with other items. Calculated Cronbach alpha of the MMCL 12‐item positive (CL‐P) subscale revealed acceptable internal consistency of.79. Consistent with this, the MMCL 6‐item negative (CL‐N) subscale was also found to demonstrate acceptable internal consistency (alpha = .72).

## 4 DISCUSSION

During the perinatal period, in pregnancy and post‐natally, women are at increased risk of mood disturbance.^1^, ^2^ *Perinatal mental* illness, particularly when unidentified and untreated, has the potential to affect maternal morbidity and family well‐being substantially.^44^, ^45^, ^46^ While less common, serious mental health problems may arise. Temporary dips and fluctuations in mood may also affect women and their families, and it is important to be able to document positive changes in mood and recovery as well as negative alterations.

Mental health and mood disturbance has commonly been assessed by standard measures such as the EPDS, GAD7, PHQ9, and PHQ4.^2^, ^47^, ^48^, ^49^, ^50^ There are methodological and practical issues associated with the screening instruments used for the measurement of women's symptoms and subsequent diagnosis.^8^, ^11^ The more commonly used measures designed for use in the perinatal period are psychometrically based, but women often indicate that the items are often quite leading, allowing them to adjust their responses to how their wish to be perceived by health care professionals.^6^

The findings of the present validation study suggest that, endorsed by women's views, a short mood checklist is an effective way of assessing how women feel in the early months after giving birth. The overall factor structure of the checklist is similar to those arising from the CFA modelling with the PANAS and the SPANE in that negative and positive scales index two distinct, but moderately negatively correlated, factors,^13^, ^14^, ^51^ although there is little overlap in the terms actually used in the measures.

While measures of affect, such as the PANAS, have been used as alternatives to more lengthy questionnaires concerning mood, these were not developed specifically with women and the perinatal period in mind, nor have they been validated for use specifically with this population.

In light of these measurement constraints, there is an identified need in health care practice for an effective measure of mood in a user‐friendly short‐form instrument with high face validity that has been developed with and for women during the perinatal period. In this validation study, the MMCL shows its ability to discriminate between women with depressive symptoms and non‐depressed women as measured by the EPDS, where caseness is set at the accepted clinically significant level of a score of 13 or greater. The instrument, which could be used as a screener, is sensitive to detecting cases, giving clinicians a potential tool that is easy for women to complete and that is easily scored.

However, the factor structure will be reassessed in further development of the scale to ensure that a two‐factor structure remains the optimal solution. This will serve to further confirm the decision to remove the third factor on account of its low internal consistency and significant decrease of eigenvalue.

A particular strength of this work is that the measure was developed with and for the group of women we wished to be the focus of the measure. In working from the initial concept stage through interviewing women about their views of the adjectives included, the resulting measure contains terms that represent the way that real women feel during this important time of their lives. By interviewing, we gained insights into their experience of the measure completion process and learned that for them, this normalized the range of positive and negative feelings that they might experience following the birth of their baby. Key strengths of the study come from the development and validation process. The measure was deemed user‐friendly by the target population for whom it had high face validity and validation involved standard recognized factor analytic methods.

Based on a pilot survey study, the 28% response rate is a limitation of the study. Further field testing of the measure is planned with recruitment of other samples, and it is recognized that test‐retest procedures should be carried out in further scale development before it is deemed suitable for use in general practice with women in the perinatal period. At the same time, it is recognized that it would be beneficial to conduct further validation analyses where the MMCL is compared with other measures designed for a broad range of mood disorders during the perinatal period to ensure the suitability of its application prior to routine use by clinicians.

With increased understanding of the importance of the mental health and well‐being of women during the perinatal period, there is an ever‐increasing need to develop instruments that provide effective and simple methods of measurement and monitoring.^50^ Effective and accurate early identification of mood disturbances is crucial. This is particularly the case in the post‐natal period when low mood may markedly affect the developing relationship a woman has with her infant and may significantly impact the quality of other relationship relationships within the family. The MMCL was developed expressly to address these issues with assessment of this population, demonstrating good psychometric properties, providing end users with confidence in measuring what is intended to be measured, with cross‐validation with an existing commonly used measure.

## 5 CONCLUSION

The MMCL gives rise to a two‐factor model with an excellent fit to the data. The measure offers women a novel method of reporting on their mood that may help them to describe both their positive and negative feelings using an engaging format, allowing more opportunities for conversations about mood, mental health, and well‐being. Upon further scale development and refinement, health care professionals may wish use this as an additional or alternative tool that is psychometrically robust, time efficient, and has the potential to afford them greater qualitative insight in the emotional state of the women they care for as it changes over time.

## CONFLICT OF INTEREST

The authors declare they have no conflict of interest.
